# Dietary exposure to aflatoxin B1, aflatoxin G1, ochratoxin A, and patulin through fruit juice consumption: A probabilistic assessment of health risk

**DOI:** 10.1016/j.toxrep.2025.101894

**Published:** 2025-01-04

**Authors:** Seyedeh Faezeh Taghizadeh, Ghazaleh Tabriznia Tabrizi, Hamid Ahmadpourmir, Gholamreza Karimi, Ramin Rezaee

**Affiliations:** a Applied Biomedical Research Center, Mashhad University of Medical Sciences, Mashhad, Iran; b Student Research Committee, Mashhad University of Medical Sciences, Mashhad, Iran; c Medical Toxicology Research Center, Faculty of Medicine, Mashhad University of Medical Sciences, Mashhad, Iran; d Department of Pharmacodynamics and Toxicology, School of Pharmacy, Mashhad University of Medical Sciences, Mashhad, Iran

**Keywords:** Mycotoxins, Cancer risk, Co-occurrence, Microbial contamination, Monte Carlo Simulation

## Abstract

The present investigation assessed the risk of dietary exposure to four mycotoxins, namely aflatoxin B1 (AFB1), aflatoxin G1 (AFG1), ochratoxin A (OTA), and patulin (PAT) via fruit juice consumption for Iranian consumers. In 96 fruit juice samples obtained from Iran market, mycotoxins levels were determined using liquid chromatography-tandem mass spectrometry. Also, probabilistic health risk assessment was conducted in terms of tolerable daily intake percentage (%TDI) and under cancer risk scenarios. The average concentrations of mycotoxins in the fruit juice samples did not vary significantly among the analyzed samples. The highest mean total level of AFB1 and AFG1was observed in sour cherry, and that of OTA and PAT in pomegranate and apple juice samples. The sour cherry juice demonstrated the highest %TDIs for AFB1 and AFG1 at 50th, 80th, and 95th centiles, while pomegranate juice and apple juice were associated with the highest %TDIs for OTA and PAT, respectively. Across all fruit juice samples, %TDIs for PAT remained below 1.0 at the three centiles. However, %TDIs for AFB1, AFG1, and OTA exceeded 1.0 at these centiles. Based on Monte Carlo Simulation model used for cancer risk scenario, at these centiles, oral consumption of the analyzed samples poses no carcinogenic risk for exposure to AFB1 and AFG1.

## Graphical Abstract

**Figure:**
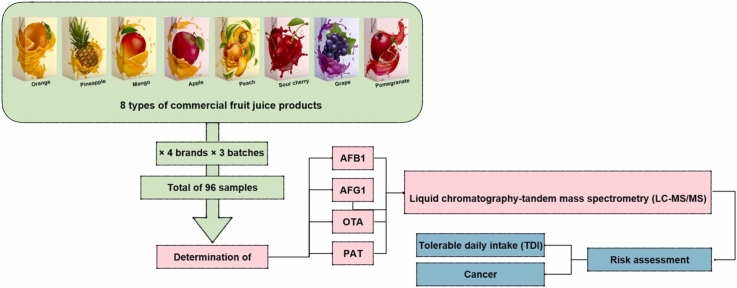

## 1 Introduction

Mycotoxins (MTs) namely, aflatoxins (AFs), ochratoxins (OTs), fumonisins (FUMs), deoxynivalenol (DON), zearalenone (ZEN), citrinin (CIT), sterigmatocystin (STE), cyclopiazonic acid (CPA), patulin (PAT), gliotoxin (GLI), and T-2 toxin are secondary metabolites generated by aerobic, microscopic fungi under specific conditions of moisture and temperature. Certain fungi establish colonies in various food items from the point of harvest to the retail stage. In recent years, MTs have attracted global attention concerning their impact on human health. This has led to heightened awareness and the implementation of stringent regulations concerning the permissible levels of MTs in food products [1], [2]. Chronic effects of MTs include heptatotoxic (AFs), oestrogenic (ZEN), immuno/haematotoxic (PAT and trichothecenes, FUMs), dermonecrotic (trichothecenes), nephrotoxic (OTA) or neurotoxic (tremorgenic toxins) effects [3]. According to the International Agency for Research on Cancer (IARC), some MTs including AFB1 and OTA are carcinogenic [4].

Various factors that influence the level of MTs in fruits are the type and cultivar, the geographical location of their cultivation and harvesting, the prevailing climatic conditions, pre-harvest practices, harvest methodology, presence of surface imperfections on the fruits, post-harvest procedures, and storage environment. The accumulation of MTs in fruits can transpire at different stages including field cultivation, harvesting, postharvest handling, and storage. Despite attempts to regulate fungal contamination in food products, fungi that produce MTs are prevalent natural contaminants and are able to infiltrate fruits either while they are still in the field or orchard, or at any stage of the harvesting, processing, storage, and distribution process. While most food items possess chemical and/or physical characteristics that make them prone to spoilage by fungi or microbes, fruits are particularly vulnerable to fungal spoilage rather than microbial spoilage due to their high-water activity and sugar content [5]. Fruit juices represent a valuable reservoir of antioxidants, vitamins, and minerals that contribute significantly to the mitigation of cardiovascular ailments, cancer, and diabetes. Fruit juices are particularly favored by minors and individuals pursuing nutritious dietary patterns, owing to their freshness, rich vitamin composition, and minimal caloric content [6]. Nevertheless, they possess nutrients that contribute to the growth of molds, yeasts, and bacteria that can withstand acidic environments. The most commonly-found MTs in fruits and fruit juices are AFs, ochratoxin A (OTA), and PAT [7].

The pathogenic fungus *Aspergillus flavus* has demonstrated significant virulence on mature fruits. The susceptibility of fresh fruits to *A. flavus* infection occurs not only on the surface but also internally through conidia transported by insects. A study revealed that immature fruits exhibit resistance to *A. flavus* invasion, whereas ripening and softening fruits lose this resistance [8]. Fruits serve as a suitable substrate for the production of AFs due to their elevated levels of carbohydrates, a characteristic that promotes toxin generation over mold proliferation. It was also observed that spores present on the surface of mature fruits could successfully initiate infection and colonization, indicating the penetration capacity of *A. flavus* conidia through the fruit skin as opposed to entry through a wound [8].

PAT is a MTs that is synthesized by certain strains of *Penicillium* and *Byssochlamys* fungi, displaying various harmful effects such as nephrotoxic and immune-toxic effects, antibiotic resistance, carcinogenicity, and mutagenicity. It has a high solubility in water and remains stable in acidic conditions, allowing it to predominantly contaminate apple-based products like juices. A previous study reported that pasteurized juices may contain PAT due to the presence of certain *Byssochlamys* species that can withstand the heat treatment commonly used in fruit juice processing [9].

OTA is a toxic substance produced by certain types of *Aspergillus* and *Penicillium* fungi, and it has harmful effects on the kidneys, and can be carcinogenic, teratogenic, genotoxic, and potentially neurotoxic. The presence of OTA in fruit juices is due to improper agricultural and harvesting practices, particularly when there is physical or physiological damage to the fruits [9]. Environmental factors like climate can also impact the production of OTA by fungi and the overall levels of OTA. OTA is commonly found in subtropical and temperate regions, and it can be detected in a variety of food products from these areas, such as grape products [9]. Similar to other MTs, OTA can withstand high temperatures during food processing, but some of it may be eliminated during fermentation [9].

Regular monitoring of food is important for ensuring food safety. In a previous study on the occurrence of contaminants in fruit juice samples, we analyzed 96 fruit juice samples for heavy metals and pesticides [10]. In the current research, we collected another set of 96 fruit juice samples from Iran market, analyzed them for the presence of MTs (AFB1, AFG1, OTA, and PAT), and finally, conducted a probabilistic risk assessment for Iranian consumers.

## 2 Materials and methods

### 2.1 Chemicals and standards

The standards of AFB1, AFG1, OTA, and PAT were purchased from Sigma Aldrich. Stock solutions of 100 mg/L were prepared in methanol and working solutions were obtained by diluting the stock solutions. All solutions were stored in darkness at −18 ºC. Solvents (acetonitrile, ethyl acetate, methanol, and chloroform) were supplied by Merck (Darmstadt, Germany).

### 2.2 Sample collection

**Fig. 1:**
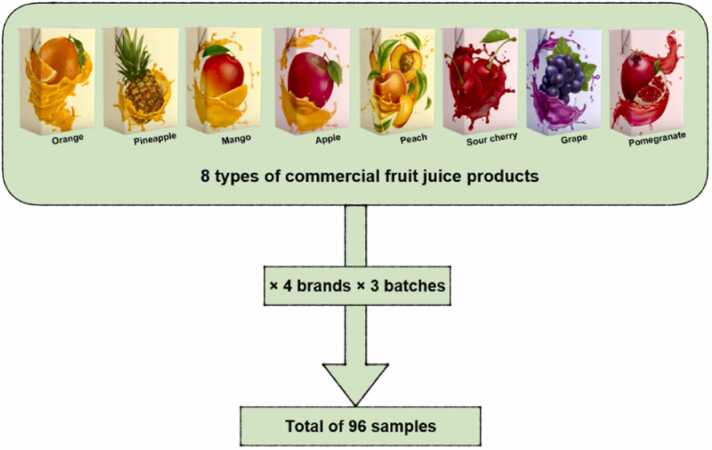
Schematic presentation of samples collection process.

A total of 96 samples of different types (orange, pineapple, mango, apple, peach, sour cherry, grape, and pomegranate) of commercial fruit juice products (Tetra Pak® packaging) were purchased from 2023 to 2024 and transported to the lab under refrigeration at 4 ºC (Fig. 1).

### 2.3 Dispersive liquid-liquid microextraction procedure (DLLME)

The development of DLLME was carried out to achieve the miniaturization, simplification, and automation of the analytical process, thus producing favorable outcomes. DLLME constitutes a ternary system comprising an aqueous solution, an organic extraction solvent (commonly a solvent possessing high density), and a dispersive solvent (miscible in both the extractant and aqueous phases). The utilization of DLLME has demonstrated several benefits, including high recovery rates and cost-effective applications [6], [11], [12], [13]. In our study, the fruit juices were extracted according to Pallarés et al. [6] and Carballo et al. [14]. From each sample, 5.0 mL was added to 1.0 g NaCl and vortexed for 1.0 min. Subsequently, 950 µL of acetonitrile (dispersion solvent) and 620 µL of ethyl acetate (extraction solvent) were added and vortexed for 1.0 min, resulting in a cloudy solution of the three components. The resultant mixture underwent centrifugation at 4000 rpm for a period of 5 min, leading to the isolation of 600 µL of the upper organic layer which was then transferred to a separate tube. In the subsequent step, 950 µL of methanol (dispersion solvent) and 620 µL of chloroform (extraction solvent) were added to the residual content within the tube. Following centrifugation, 600 µL of the lower organic layer was retrieved and amalgamated with the previously separated organic phase. The two retrieved phases were subsequently evaporated until near dryness, using a nitrogen stream [6], [14].

### 2.4 Liquid chromatography-tandem mass spectrometry (LC-MS/MS) determination

AFB1, AFG1, OTA, and PAT were identified and quantified by an Agilent 1200 LC (Agilent Technologies, USA) coupled with 3200 QTRAP® MS equipped with electrospray ionization interface turbo ion spray (ESI). A Gemini NX-C18 column (150 ×2 mm, particle size 3 μm, 110 A) preceded by a C18 column was used for analytes separation. The duration of the experiment was 30 min with an injection volume of 20 μL. The mobile phase consisted of eluent A (H_2_O + 5 mM ammonium formate + 0.1 % formic acid) and eluent B (Methanol + 5 mM ammonium formate + 0.1 % formic acid). The flow rate was adjusted to 0.400 mL/min with eluent B at 100 %. Subsequently, between min 21 and 30, the flow rate was reduced to 0.250 mL/min with 10 % eluent B and 90 % eluent A. The key characteristics of the mass spectrometry setup included multiple reaction monitoring, ion source turbo spray, and positive ionization polarity (ESI+) [6].

### 2.5 Method validation

The method was validated in terms of recoveries, matrix effect (ME), limit of detection (LOD) and limit of quantification (LOQ) according to the European Commission [15]. The recoveries were obtained by spiking blank juice samples with the concentration levels of 0.005, 0.01, and 0.02 mg/kg for AFB1, AFG1, and OTA at and 0.25, 0.50, and 1.00 mg/kg for PAT in three replicates. In most multi-residue analyses utilizing mass detection, variations in signal intensity of an analyte dissolved in different solvents can be observed. Consequently, a study on ME was conducted to assess the precision of the analytical method using the Eq. 1 [6], [16].

(1) $$\mathrm{Matrix effect}\left(\%\right)=\left(\frac{\mathrm{Slope of calibration curve for matrix}}{\mathrm{Slope of calibration curve for solvent}}\;-1\right)\;\times 100$$

*Positive ME value: Signal increment (Higher signal in the matrix than in the solvent)*

*Negative ME value: Signal Suppression (Lower signal in the matrix than in the solvent)*

*Zero ME value: Matrix and solvent calibration graphs have the same slope*

*Low ME: 0–20 %, medium ME: 20–50 % and strong ME: ˃50 %*

The LODs and LOQs were established by adding known amounts of MTs to blank juice samples, with concentrations gradually decreasing. The criterion for determining both transitions was set at a signal-to-noise ratio (S/N) of at least 3 for calculating the LOD and 10 for the LOQ [17].

### 2.6 Health risk assessment

Estimated daily intake (EDI) for chronic dietary exposure to AFB1, AFG1, OTA, and PAT via ingestion of the analyzed fruit juice samples was calculated by Eq. 2 [18].

(2) $$\mathrm{EDI}=\frac{\mathrm{IR}\times C\;\times \mathrm{ED}\times \mathrm{EF}}{\mathrm{BW}\times \mathrm{AT}}$$

*IR: Ingestion rate, the daily date consumption (kg) (0.03 kg or L/person/day for Iranian general population)* [10] *.*

*C: Each of AFB1, AFG1, OTA, and PAT concentration in fruit juice (mg/kg)*

*ED: Exposure duration for elders (70 years); EF: Exposure frequency (365 meals/year); BW: Average body weight for Iranian adult population is considered 70 kg; AT: Average time (25,550 days or 70 years)* [19] *.*

Health risk of exposure to these four toxins via juice consumption was assessed in terms of percent of tolerable daily intake (TDI) (Eq. 3) and the cancer risk (Eq. 4) [20].

(3) $$\%\mathrm{TDI}=\;\left(\frac{\mathrm{EDI}}{\mathrm{TDI}}\right)\;\times 100$$

*TDI: Tolerable daily intake*

*TDI for: AFB1 and AFG1 = 0.001 µg/kg bw; OTA= 0.016 µg/kg bw; PAT= 0.40 µg/kg bw* [4], [21] *.*

Cancer risk was estimated only for AFB1 and AFG1 (Group 1 members (i.e. carcinogenic to humans)) as per IARC classification [4], [22].

(4) Cancer risk=EDI×CPS

*CPS: The carcinogenic potency slope for ingestion: 0.3 and 0.01 cancers per year per 10* ^*5*^ *population per ng of AFB1 and AFG1 per kg bw per day, respectively for people with a positive surface antigen of hepatitis B (HBsAg* ^*+*^ *) and for people with a negative surface antigen of hepatitis B (HBsAg* ^*-*^ *).*

Exposure assessment of AFs has experienced significant development over the last twenty years, primarily attributed to the identification of biomarkers for both exposure and impact of these toxins. Garba et al. reported that the IPSC/WHO aflatoxin risk assessment utilized distinct cancer potency factors for aflatoxin: 0.01 cases/100,000/year/ng/kg body weight per day for individuals without chronic HBV infection, and 0.30 cases for those with chronic HBV infection. This decision stemmed from a cohort study that evaluated cancer potency in HBV surface antigen (HBsAg) positive individuals (a biomarker of chronic HBV infection) and HBsAg negative individuals, alongside other research studies that investigated cancer potency in either HBsAg-positive or HBsAg-negative individuals [23]. Nonetheless, numerous epidemiological studies have verified that the cancer potency of aflatoxins is approximately 30 times higher in HBV-positive individuals compared to HBV negative ones [23], [24].

### 2.7 Probabilistic and statistical calculations

A Monte Carlo simulation (MCS) was run for the assessment of the distributions pertaining to the variables. The examination encompassed 10,000 iterations concerning the risks associated with the oral exposure to fruit juice samples. The evaluation of risk and uncertainty was conducted utilizing JMP 8 software (Campus Drive, Cary, NC 27513). The statistical analysis of the mean concentration of MTs in the fruit juice samples collected was performed using GraphPad Prism 8.0 (GraphPad Software, San Diego, CA, USA). A post-test was utilized for the comparison of means of parametric data. The threshold for statistical significance was established at a *p-*value less than 0.05 [25].

## 3 Results

### 3.1 Analytical performance

The mean percent of recovery for the MTs ranged from 93.50 % to 99.1 %, with an associated Relative Standard Deviation (RSD) of ≤ 7.3 % (Table 1). The correlation coefficients (R^2^) were 99.7–99.8 %. The LODs and LOQs ranges were 0.0006–0.0017 and 0.0018–0.005 mg/kg, respectively (Table 1).

**Table 1:** The analytical performance characteristics.

MTs	Spiked level			ME (%)	R^2^	LOD (mg/kg)	LOQ (mg/kg)
	0.005 (mg/kg)	0.01 (mg/kg)	0.02 (mg/kg)				
AFB1	Mean recovery (%) ± RSD				0.998	0.0013	0.0038
	95.5 ± 5.2	97.3 ± 3.2	99.1 ± 2.5	48.0			
AFG1	95.2 ± 5.0	97.0 ± 3.2	98.5 ± 2.5	55.0	0.998	0.0013	0.0038
OTA	94.0 ± 6.5	96.5 ± 4.3	98.1 ± 3.0	60.0	0.997 0.997	0.0006 0.0017	0.0018 0.0050
PAT	0.25 (mg/kg)	0.5 (mg/kg)	1.00 (mg/kg)				
	93.5 ± 7.3	95.1 ± 5.5	97.3 ± 3.0	68.0			

MTs: Mycotoxin; RSD: Relative Standard Deviation; AFB1: Aflatoxin B1; AFG1: Aflatoxin G1; OTA: Ochratoxin A; PAT: Patulin; ME: Matrix effect; R^2^: Correlation coefficients; LOD: Limit of Detection; LOQ: Limit of Quantitation.

### 3.2 Concentrations of mycotoxins (MTs)

The mean concentrations of the MTs in the analyzed fruit juice samples are shown in Table 2. The substitution method is predominantly utilized by the European Food Safety Authority (EFSA) for handling results that fall below the LOD. In this approach, the analytical value is replaced by the LOD itself, either as the upper bound, lower bound (zero), or as the middle bound (LOD/2) [26]. The upper bound was employed in the current investigation. Among the analyzed fruit juice samples, the levels of MTs did not exhibit significant variations (Table 2). The highest mean total AFB1 and AFG1 levels in fruit juice samples were observed in sour cherry juice (0.0063 and 0.0041 mg/kg, respectively). In pomegranate and apple juice, the mean total OTA and PAT were 0.0046 and 0.0066 mg/kg, respectively.

**Table 2:** Mean total concentration of mycotoxins (mg/kg) in fruit juice samples.

Product	AFB1	AFG1	OTA	PAT
Apple juice	0.0031 ± 0.001	0.0033 ± 0.001	0.0021 ± 0.000	0.0066 ± 0.001
Grape juice	0.0022 ± 0.000	0.0016 ± 0.000	0.0010 ± 0.000	0.0025 ± 0.000
Mango juice	0.0022 ± 0.000	0.0024 ± 0.001	0.0010 ± 0.000	0.0028 ± 0.000
Orange juice	0.0040 ± 0.001	0.0022 ± 0.000	0.0024 ± 0.000	0.0055 ± 0.000
Peach juice	0.0040 ± 0.001	0.0013 ± 0.000	0.0022 ± 0.000	0.0053 ± 0.001
Pineapple juice	0.0027 ± 0.001	0.0013 ± 0.000	0.0011 ± 0.000	0.0045 ± 0.001
Pomegranate juice	0.0056 ± 0.001	0.0030 ± 0.000	0.0046 ± 0.001	0.0042 ± 0.001
Sour cherry juice	0.0063 ± 0.001	0.0041 ± 0.001	0.0022 ± 0.000	0.0030 ± 0.000

AFB1: Aflatoxin B1; AFG1: Aflatoxin G1; OTA: Ochratoxin A; PAT: Patulin

### 3.3 Health risk assessment

The percentages of the TDIs at the 50th, 80th, and 95th centiles, as produced by the MCS, for fruit juice samples are presented in Table 3. Sour cherry juice exhibited the highest %TDIs (SUM) at the three centiles. Conversely, grape juice displayed the lowest %TDIs (SUM) at these centiles. Sour cherry juice demonstrated the highest %TDIs for AFB1 and AFG1 at the three centiles, while pomegranate juice and apple juice were associated with the highest %TDIs for OTA and PAT, respectively (Table 3). Across all fruit juice samples, the %TDIs for PAT remained below 1.0 at all three centiles. However, the %TDIs for AFB1, AFG1, and OTA exceeded 1.0 at the 50th, 80th, and 95th centiles (Table 3).

**Table 3:** Percent of tolerable daily intake (%TDI) calculated using Monte Carlo Simulation for fruit juice samples.

Product	AFB1	AFG1	OTA	PAT	SUM
**%TDI (50th)**					
Apple juice	71.815	77.600	3.194	0.460	153.070
Grape juice	52.722	38.258	1.591	0.217	92.788
Mango juice	52.915	56.000	1.506	0.235	110.658
Orange juice	93.993	52.722	3.555	0.392	150.664
Peach juice	90.330	30.158	3.387	0.380	124.253
Pineapple juice	63.910	30.158	1.687	0.337	96.0912
Pomegranate juice	130.058	67.765	6.786	0.318	204.927
Sour cherry juice	147.415	95.730	3.362	0.244	246.752
**%TDI (80th)**					
Apple juice	110.382	119.275	4.910	0.705	235.273
Grape juice	81.036	58.804	2.445	0.333	142.620
Mango juice	81.332	86.075	2.315	0.362	170.086
Orange juice	144.471	81.036	5.465	0.604	231.577
Peach juice	138.840	46.354	5.206	0.582	190.982
Pineapple juice	98.230	46.354	2.593	0.518	147.695
Pomegranate juice	199.904	104.157	10.430	0.490	314.981
Sour cherry juice	226.582	147.140	5.170	0.375	379.267
**%TDI (95th)**					
Apple juice	132.723	143.437	5.647	0.582	282.390
Grape juice	97.366	70.580	2.678	0.133	170.758
Mango juice	97.723	103.437	2.522	0.170	203.851
Orange juice	173.794	97.366	6.316	0.460	277.937
Peach juice	167.010	55.580	6.004	0.433	229.027
Pineapple juice	118.080	55.580	2.857	0.357	176.874
Pomegranate juice	240.580	125.223	12.300	0.321	378.423
Sour cherry juice	272.723	177.010	5.960	0.184	455.876

AFB1: Aflatoxin B1; AFG1: Aflatoxin G1; OTA: Ochratoxin A; PAT: Patulin

The cancer risks for HBsAg^+^ and HBsAg^-^ are shown in Table 4. In fruit juice samples, the maximum and minimum cancer risks for total HBsAg^+^ and HBsAg^-^ were observed for sour cherry and grape juices, respectively. For sour cherry juice, the risks for total HBsAg^+^ at the 50th, 80th, and 95th centiles were 5.884E-07, 9.550E-07, and 1.088E-06, respectively. The risks for total HBsAg^-^ were 2.253E-08, 3.328E-08, and 4.326E-08 for sour cherry juice. Moreover, at the 50th, 80th, and 95th centiles, the cancer risks for total HBsAg^+^ in grape juice were respectively 2.200E-07, 3.570E-07, and 4.070E-07, and for total HBsAg^-^ were 8.424E-09, 1.244E-08, and 1.617E-08, respectively (Table 4).

**Table 4:** Cancer risk calculated using Monte Carlo Simulation for fruit juice samples.

Product	AFB1	AFG1	SUM	AFB1	AFG1	SUM
	(HBsAg^+^)	(HBsAg^+^)	(HBsAg^+^)	(HBsAg^-^)	(HBsAg^-^)	(HBsAg^-^)
**50th**						
Apple juice	1.737E−07	1.877E−07	3.615E−07	6.653E−09	7.190E−09	1.384E−08
Grape juice	1.275E−07	9.247E−08	2.200E−07	4.882E−09	3.541E−09	8.424E−09
Mango juice	1.280E−07	1.354E−07	2.634E−07	4.900E−09	5.186E−09	1.010E−08
Orange juice	2.274E−07	1.275E−07	3.550E−07	8.710E−09	4.882E−09	1.360E−08
Peach juice	2.185E−07	7.286E−08	2.914E−07	8.370E−09	2.790E−09	1.116E−08
Pineapple juice	1.545E−07	7.286E−08	2.274E−07	5.920E−09	2.790E−09	8.710E−09
Pomegranate juice	3.147E−07	1.640E−07	4.787E−07	1.205E−08	6.277E−09	1.833E−08
Sour cherry juice	3.568E−07	2.316E−07	5.884E−07	1.366E−08	8.871E−09	2.253E−08
**80th**						
Apple juice	2.820E−07	3.046E−07	5.866E−07	9.826E−09	1.061E−08	2.044E−08
Grape juice	2.070E−07	1.500E−07	3.570E−07	7.211E−09	5.230E−09	1.244E−08
Mango juice	2.076E−07	2.198E−07	4.274E−07	7.237E−09	7.660E−09	1.490E−08
Orange juice	3.691E−07	2.070E−07	5.760E−07	1.286E−08	7.211E−09	2.010E−08
Peach juice	3.547E−07	1.182E−07	4.730E−07	1.236E−08	4.120E−09	1.648E−08
Pineapple juice	2.508E−07	1.182E−07	3.691E−07	8.743E−09	4.120E−09	1.286E−08
Pomegranate juice	5.108E−07	2.660E−07	7.768E−07	1.780E−08	9.271E−09	2.710E−08
Sour cherry juice	5.790E−07	3.760E−07	9.550E−07	2.018E−08	1.310E−08	3.328E−08
**95th**						
Apple juice	3.214E−07	3.473E−07	6.687E−07	1.277E−08	1.380E−08	2.657E−08
Grape juice	2.358E−07	1.710E−07	4.070E−07	9.375E−09	6.800E−09	1.617E−08
Mango juice	2.367E−07	2.505E−07	4.873E−07	9.410E−09	9.958E−09	1.936E−08
Orange juice	4.207E−07	2.358E−07	6.566E−07	1.672E−08	9.375E−09	2.610E−08
Peach juice	4.043E−07	1.347E−07	5.391E−07	1.610E−08	5.357E−09	2.142E−08
Pineapple juice	2.860E−07	1.347E−07	4.207E−07	1.136E−08	5.357E−09	1.672E−08
Pomegranate juice	5.823E−07	3.032E−07	8.856E−07	2.314E−08	1.205E−08	3.520E−08
Sour cherry juice	6.601E−07	4.285E−07	1.088E−06	2.623E−08	1.703E−08	4.326E−08

AFB1: Aflatoxin B1; AFG1: Aflatoxin G1; OTA: Ochratoxin A; PAT: Patulin

HBsAg+ : People with a positive surface antigen of hepatitis B

HBsAg-: People with a negative surface antigen of hepatitis B

## 4 Discussion

It is imperative to handle fruit meticulously and hygienically during harvesting as well as in storage and processing facilities to mitigate fungal spoilage and MTs occurrence. Processed fruit derivatives could potentially contain substantial levels of MTs if spoiled or mold-infested fruits are not eliminated prior to processing or packaging [27], [28]. The levels of MTs in the fruit juice samples examined in this investigation did not demonstrate significant variability among the eight types of juice examined. Sour cherry juice exhibited the highest average total concentrations of AFB1 and AFG1 while the highest mean total concentrations of OTA and PAT were recorded in pomegranate and apple juice, respectively. According to the MCS model, grape juice showed the lowest %TDIs (SUM) at the 50th, 80th, and 95th centiles. The highest %TDIs for AFB1 and AFG1 at these centiles were observed for sour cherry juice, whereas pomegranate juice and apple juice showed the highest %TDIs for OTA and PAT, respectively. In all fruit juice samples, at the three centiles, while the %TDIs for PAT remained below 1.0, these values surpassed 1.0 for AFB1, AFG1, and OTA. The cancer risks associated with HBsAg^+^ and HBsAg^-^ indicated that among fruit juice samples, sour cherry and grape juices exhibited the maximum and minimum cancer risks for total HBsAg+ and HBsAg-, respectively. Our cancer risk assessment for AFB1 and AFG1 indicated that at the 50th, 80th, and 95th centiles, oral intake of the samples under analysis does not present any carcinogenic risk.

The metabolites of MTs may accumulate in the liver, kidney, and other bodily organs before excretion. The presence of these metabolites in the edible tissues of animals within the food supply chain could potentially create a public health risk. In reality, individuals are exposed to a combination of chemicals (i.e. chemical mixtures) by consuming food and water. Human beings are consistently exposed to a diverse range of MTs through oral ingestion. Generally, potential health-promoting and chronic disease-preventing properties of fruit juice have led to increased public tendency towards consumption of these products; thus, regular monitoring of the occurrence of potential contaminants in fruit juice products is highly important [6], [17].

Also, to reduce the occurrence of MTs, preventative agricultural measures (including good agricultural practices, selecting appropriate varieties, cultivation methods, and plant protection treatments) need to be implemented and harvesting and storage, and conditions need to be improved. Organic farming prohibits the use of fungicides but emphasizes application of methods that diminish MTs contamination such as crop rotation, tillage, and crop history. While there is limited data on MTs contamination in organic products, varying contamination levels have been reported [29].

High acidity is a trait that most fruits have in common. Fruits have pHs between 5.0 and 2.5, and it is thought that this pH range is the most significant factor in determining the kinds of microbes that can contaminate fruits. It was shown that pH has a great influence on the aflatoxin degradation; however, the foods with low acidity were also observed to be contaminated with aflatoxin. Fungi are the main bacteria that cause fruit and fruit product deterioration because of their acidity [30]. Pallarés et al., 2019 screened 80 samples of fruit juice comprising of mono-fruit juices and blended beverages, from a Spanish retail market and among the 30 analyzed MTs, they found that AFB1, OTA, and PAT were among the 9 quantifiable toxins at the maximum concentration levels of 18.1, 10.81, and 50.95 µg/L, with the incidence percentage of 4, 9, and 18 %, respectively. Authors indicated that TDI values were reached for children exposure upon daily consumption of 200 mL of fruit juice [6]. A study by Carballo et al. reported that among 42 samples collected from Spain, PAT was detected in orange, apple, pineapple and mixed fruit juices with 86 %, 60 %, 14 %, and 29 % prevalence and mean concentrations of 34.57, 33.41, 8.02, and 8.59 µg/L, respectively. While only in one orange juice sample, PAT level (50.95 µg/L) exceeded the maximum level of 50 µg/L established by the EU Commission, it was not detected in samples of apricot and pear juice. This study reports no toxicological concern vis-à-vis mycotoxin intake through fruit juice consumption among children and adults [14]. A study from India reported PAT levels in branded and vendor-obtained apple juice samples. Their results showed that some samples had high PAT levels (845 µg/L) compared to other branded mixed juices (21–70 µg/L) and apple juices procured from local vendors (191 µg/L) [31]. These concentrations nonetheless were noticeably higher than the maximum permitted concentration (MPC) of 50 ppb for PAT established by the USFDA [32]. However, the average intake of PAT through fruit juice consumption was within the provisional maximum tolerable daily intake (PMTDI) of 0.4 µg/kg bw/day set by the Joint FAO/WHO Expert Committee on Food Additives (JECFA) [33]. In this report, researchers attributed high levels of PAT to sub-optimal manufacturing procedures involved in the production of juices where damaged apple fruits are not removed prior to the processing. Furthermore, the authors suggested a number of solutions to mitigate the overall PAT content in juice samples including the addition of sulfur dioxide, pasteurization, and removing rotten tissues from the apples [31]. A similar investigation in Italy was conducted on 105 samples of apple, mixed-taste, and pear juices [34]. Apple juice samples contained the lowest concentration of PAT (mean value 18 and maximum value 30 µg/L), followed by mixed fruit juices at (mean value 23 and maximum value 45 µg/L), and pear juices (mean value 43 and maximum value 92 µg/L). Except the pear juice samples (among which, 14 out of 35 contained PAT above the highest regulated limit of 50 µg/L (established by European Commission Regulation 1881/06)), other juice samples were considered safe with regard to PAT contamination. Furthermore, an inverse association between the fruit content of the juices and the PAT contamination was reported. Juice samples with less than 50 % fruit content contained average PAT levels of 31.55 µg/L while samples with higher proportions of fruit contained an average PAT level of 23.41 µg/L. Researchers attributed this observation to the use of low-quality raw materials and involvement of dilution [34]. Mango juice samples collected from Pakistan were determined to contain relatively high levels of PAT (mean value 24.3 µg/kg and maximum value 226 µg/kg) with a high incidence of 70 % and in the majority of samples, PAT level exceeded the regulatory limit of 50 µg/kg; in this study, maximum level of PAT in orange juice sample was 31 µg/kg [35]. A report from Turkey, determined mean PAT levels of 733 µg/kg in apple juice samples [36].

Generally, AFs, PAT, and OTA occurrence has been reported in fruits and their processed derivatives including fruit juices from different countries. These toxins even at low concentrations, particularly when the exposure is chronic, can pose a serious risk to human health [37], [38]. There is a growing concern about the presence of MTs in fruits and their processed products. Of note, children, who more-frequently consume large quantities of fruit juices, are particularly at risk. Continuous monitoring programs, especially at national levels, are needed to safeguard consumers from exposure to unsafe levels of MTs in fruit juice products.

## 5 Conclusion

The mean concentrations of MTs in the fruit juice samples did not show any statistically significant variation among different samples. Sour cherry juice exhibited the highest average levels of total AFB1 and AFG1 and pomegranate and apple juice had respectively the highest mean levels of OTA and PAT among the fruit juice samples. AFB1 and AFG1 had the highest %TDIs in sour cherry juice at all three centiles, while pomegranate juice and apple juice showed the highest %TDIs for OTA and PAT. While for all samples, at the 50th, 80th, and 95th centiles, %TDIs for PAT remained below 1.0 these values surpassed 1.0 for AFB1, AFG1, and OTA. Based on a Monte Carlo Simulation run for cancer risk scenario, at the 50th, 80th, and 95th centiles, oral consumption of the analyzed samples poses no carcinogenic risk for exposure to AFB1 and AFG1. It is important to consider that consumers are daily also exposed to MTs through other foodstuffs and other routes of exposure. To ensure consumers safety regarding exposure to MTs via fruit juice consumption, it is essential to regularly monitor the MTs levels and compare the contamination concentrations with internationally accepted limits. Also, research is required to discern fruit cultivars that exhibit resistance to fungal decay and develop treatment approaches for managing fungal pathogens in both pre-harvest and post-harvest stages.

## Funding

This work was financially supported by grants from the 10.13039/501100004748Mashhad University of Medical Sciences, Mashhad, Iran [Project number 4011935]. Also, the ethics committee of Mashhad University of Medical Sciences, Mashhad, Iran approved execution of the present study.

## Ethical statement

In Acknowledgement thankful to Vice Chancellor of Research, Mashhad University of Medical Sciences, Mashhad, Iran, for the financial support (Grant No. 4011935 and Ethics committee approval No. IR.MUMS.REC.1401.410).

## CRediT authorship contribution statement

**Gholamreza Karimi:** Validation, Project administration, Investigation, Conceptualization. **Ramin Rezaee:** Writing – review & editing, Supervision, Funding acquisition. **Ghazaleh Tabriznia Tabrizi:** Resources, Methodology, Investigation, Data curation. **Hamid Ahmadpourmir:** Visualization, Resources, Investigation, Data curation. **Seyedeh Faezeh Taghizadeh:** Writing – original draft, Software, Methodology, Formal analysis, Data curation.

## Declaration of Competing Interest

Authors declare that there is no conflict of interest.

## Data Availability

Data will be made available on request.
